# NSAID–Gut Microbiota Interactions

**DOI:** 10.3389/fphar.2020.01153

**Published:** 2020-08-07

**Authors:** Damian Maseda, Emanuela Ricciotti

**Affiliations:** ^1^ Department of Microbiology, University of Pennsylvania, Philadelphia, PA, United States; ^2^ Department of Systems Pharmacology and Translational Therapeutics, and Institute for Translational Medicine and Therapeutics, University of Pennsylvania, Philadelphia, PA, United States

**Keywords:** NSAIDS (nonsteroidal anti-inflammatory drugs), microbiota (microorganisms), dysbiosis, enteropathy, prostanoid, cyclooxygenase

## Abstract

Nonsteroidal anti-inflammatory drugs (NSAID)s relieve pain, inflammation, and fever by inhibiting the activity of cyclooxygenase isozymes (COX-1 and COX-2). Despite their clinical efficacy, NSAIDs can cause gastrointestinal (GI) and cardiovascular (CV) complications. Moreover, NSAID use is characterized by a remarkable individual variability in the extent of COX isozyme inhibition, therapeutic efficacy, and incidence of adverse effects. The interaction between the gut microbiota and host has emerged as a key player in modulating host physiology, gut microbiota-related disorders, and metabolism of xenobiotics. Indeed, host–gut microbiota dynamic interactions influence NSAID disposition, therapeutic efficacy, and toxicity. The gut microbiota can directly cause chemical modifications of the NSAID or can indirectly influence its absorption or metabolism by regulating host metabolic enzymes or processes, which may have consequences for drug pharmacokinetic and pharmacodynamic properties. NSAID itself can directly impact the composition and function of the gut microbiota or indirectly alter the physiological properties or functions of the host which may, in turn, precipitate in dysbiosis. Thus, the complex interconnectedness between host–gut microbiota and drug may contribute to the variability in NSAID response and ultimately influence the outcome of NSAID therapy. Herein, we review the interplay between host–gut microbiota and NSAID and its consequences for both drug efficacy and toxicity, mainly in the GI tract. In addition, we highlight progress towards microbiota-based intervention to reduce NSAID-induced enteropathy.

## Introduction

Nonsteroidal anti-inflammatory drugs (NSAIDs) are among the most commonly used drugs worldwide for the treatment of pain, inflammation, and fever. NSAIDs exert their pharmacological effects through the inhibition of the cyclooxygenase (COX) enzyme. COX is the rate-limiting enzyme involved in the biotransformation of arachidonic acid (AA) into prostanoids, including prostaglandin (PG)E_2_, PGD_2_, PGF_2_ *_α_*, prostacyclin (PGI_2_), and thromboxane (TxA_2_). COX exists in two isozymes, known as COX-1 and COX-2, which have different physiological functions, gene regulation, and pattern of expression (Ricciotti and FitzGerald, 2011). COX-1 is constitutively expressed in almost all tissues and is responsible for the production of prostanoids that control homeostatic functions, such as gastric epithelial cytoprotection, platelet function homeostasis, and renal blood flow regulation (Ricciotti and FitzGerald, 2011). COX-2 is an immediate response gene. Its basal expression is restricted to certain organs, including the kidney, the central nervous system, and the vasculature. COX-2 gene and protein expression are rapidly induced by inflammatory cytokines, laminar shear stress, and growth factors, and it represents the main source of prostanoid formation during the inflammatory response (Ricciotti and FitzGerald, 2011).

NSAIDs are part of a chemically heterogeneous group of compounds that can be classified on the basis of their relative inhibition of COX isozymes. Based on their selectivity for COX-1 and COX-2 inhibition achieved by therapeutic doses, NSAIDs can be broadly classified into nonselective COX inhibitors, such as aspirin, ibuprofen, indomethacin, and naproxen and selective COX-2 inhibitors, such as diclofenac and coxibs (*e.g.* celecoxib, rofecoxib, *etc.*).

Despite their efficacy in the relief of pain and inflammation, NSAIDs can cause serious adverse events such as gastrointestinal (GI) and cardiovascular (CV) complications in some individuals (Grosser et al., 2017; Bjarnason et al., 2018). The coxibs, rationally designed COX-2 selective inhibitors, were originally developed to reduce the incidence of serious GI adverse effects when compared with nonselective NSAIDs (Bjarnason et al., 2018).

GI toxicity is arguably a significant adverse effect associated with NSAID use, due to its frequency and severity. Short- and long-term use of NSAIDs can cause upper and lower GI damage, predominantly in patients with predispositions (Bjarnason et al., 2018). About 30 to 50% of NSAID users have endoscopic lesions in the GI tract. The incidence rates of upper GI toxicity (gastropathy), which is manifested with gastroduodenal ulcers, range from 5 to 80% in short-term (<1 month) endoscopy studies (Bjarnason et al., 2007) and from 15 to 40% in long-term (>3 months) users (Geis et al., 1991). The incidence rate of serious upper GI complications, which include ulcers, intestinal perforation, acute bleeding, and gut stenosis ranges between 1 and 2% in chronic (>3 months) NSAID users (Sostres et al., 2013). The signs and symptoms of NSAID-induced lower GI toxicity (enteropathy), localized distal to the ligament of Triez, are usually nonspecific, often are clinically silent, and difficult to detect. New endoscopic techniques enabled diagnosis of NSAID-induced enteropathy more easily than in the past and revealed that they may be as common and serious as upper GI complications (Shin et al., 2017). NSAID-induced toxicity in the small bowel can manifest with nausea, indigestion, constipation, diarrhea, and abdominal pain. Chronic exposure to NSAID can cause mucosal erythema, mucosal erosions and breaks, sub-epithelial hemorrhages, protein loss, anemia, strictures, and ulcerations. In the long term these lesions may become more serious but rarely cause intestinal obstruction and perforation. Small intestine permeability is present in 50–70% of long-term NSAID users, while inflammation is present in 60–70% of them (Tai and McAlindon, 2018). Lower GI mucosal damage is present in approximately 30–40% of NSAID users (Maiden et al., 2005; Sostres et al., 2013). Over the past decades, NSAID-induced peptic ulcer disease and the hospitalization rates due to upper GI complications have declined (Lanas et al., 2011; Malmi et al., 2014), paralleling the prescription of modified-release and/or enteric-coated NSAIDs, widespread use of anti-secretory drugs [*e.g.* proton pump inhibitors (PPIs) and histamine 2-receptor inhibitors], and reduction in *Helicobacter pylori* prevalence. Meanwhile, the incidence of lower GI damage associated with NSAIDs has become more perceptible (Bjarnason et al., 2018). Unfortunately, current prevention strategies that reduce the extent of damage in the upper GI tract are not effective in the lower GI tract. Potential new therapeutic strategies that aim to prevent lower GI tract damage caused by NSAIDs are reported in **Table 1**.

**Table 1:** Potential therapeutic interventions to reduce NSAID-induced enteropathy.

Intervention	Specie	Reference
Antibiotics	Rat	Kent et al., 1969; Yamada et al., 1993; Uejima et al., 1996; Koga et al., 1999; Konaka et al., 1999; Leite et al., 2001; Syer et al., 2015; Fornai et al., 2016; Kim et al., 2016; Colucci et al., 2018; Zhang et al., 2019
	Mouse	Liang et al., 2015; Xiao et al., 2017
	Human	Bjarnason et al., 1992; Scarpignato et al., 2017
Hydrogen Sulfide -releasing NSAID	Rat	Wallace et al., 2010; Blackler et al., 2015
Prostaglandin E_2_ analog	Mouse	Kunikata et al., 2001; Kunikata et al., 2002
	Rat	Kunikata et al., 2001; Kunikata et al., 2002; Hatazawa et al., 2006
	Human	Bjarnason et al., 1989; Davies et al., 1993; Watanabe et al., 2008b; Fujimori et al., 2009; Kyaw et al., 2018; Taha et al., 2018
Prostaglandin E_2_ receptor 3 agonist	Mouse	Kunikata et al., 2001; Kunikata et al., 2002;
Prostaglandin E_2_ receptor 4 agonist	Mouse	Kunikata et al., 2001; Kunikata et al., 2002
	Rat	Hatazawa et al., 2006
Rebamipide	Mouse	Diao et al., 2012; Tanigawa et al., 2013; Lai et al., 2015;
	Rat	Mizoguchi et al., 2001; Kurata et al., 2015
	Human	Niwa et al., 2008; Fujimori et al., 2011; Mizukami et al., 2011; Kurokawa et al., 2014; Watanabe et al., 2015; Ota et al., 2016
*β*-glucuronidase inhibitors	Mouse	LoGuidice et al., 2012; Saitta et al., 2014
Probiotics	Rat	Kinouchi et al., 1998; Watanabe et al., 2009; Syer et al., 2015; Fornai et al., 2020
	Human	Endo et al., 2011; de Vos et al., 2017; Suzuki et al., 2017; Gotteland et al., 2001; Montalto et al., 2010; Mujagic et al., 2017

In addition to GI adverse effects, NSAIDs can cause serious CV complications. NSAIDs can raise blood pressure and cause atherothrombotic events, heart failure, arrhythmias, and sudden cardiac death (Coxib and traditional NSAID Trialists’ (CNT) Collaboration et al., 2013; Grosser et al., 2017). Randomized placebo-controlled trials have established that NSAIDs confer a CV hazard in approximately 1 to 2% of people exposed, but the absolute risk may increase with higher drug doses, frequency of use, and established CV disease (Coxib and traditional NSAID Trialists’ (CNT) Collaboration, et al., 2013; Grosser et al., 2017). The increased risk for hypertension and atherothrombotic events associated with NSAID exposure is mechanistically consistent with the inhibition COX-2 dependent formation of cardioprotective PGs (Yu et al., 2012). On other hand, NSAID may increase the risk for heart failure through a direct effect on the myocardium and vasculature remodeling and/or by altering hemodynamic stability and renal function (Patrono, 2016). The increased risk for heart failure associated with NSAID use occurs through a COX-2 dependent hazard unrelated to the degree and duration of suppression of platelet COX-1 (Coxib and traditional NSAID Trialists’ (CNT) Collaboration, et al., 2013). Thus, the risk profiles of NSAIDs can vary due to the degree to which an individual drug inhibits COX-1 or COX-2 isozymes. NSAID use is associated with interindividual variability in the extent of COX isozyme inhibition and in the occurrence of therapeutic and adverse effects (Panara et al., 1999; McAdam et al., 1999; Theken et al., 2019), which could partially be explained by pharmacokinetic variability and single-nucleotide polymorphisms (SNPs) in NSAID-metabolizing genes cytochrome (CYP) P450 isoenzymes (Karaźniewicz-Łada et al., 2009; Bae et al., 2011) and in COX genes (Fries et al., 2006; Lee et al., 2017).

The interaction between host and gut microbiota (the large community of microorganisms that resides in the gut) has emerged as a key player in modulating host physiology, gut microbiota-related disorders, and metabolism of xenobiotics (dietary components, environmental pollutants, and pharmaceuticals; Sommer and Bäckhed, 2013; Vayssier-Taussat et al., 2014; Spanogiannopoulos et al., 2016). The human gut microbiota, by modulating drug disposition, is now recognized as a novel factor driving interindividual variation in drug efficacy and side effects, including those of NSAIDs. Indeed, the heterogeneity of the gut microbiota in populations may result in different responses to drug treatment (Yip and Chan, 2015; Kashyap et al., 2017).

In this review, we discuss the field’s current knowledge of NSAID–gut microbiota interactions. We report on studies describing how NSAIDs can modify the growth and imbalance the composition of the intestinal microbial communities (condition known as dysbiosis) and the effects of these modifications on the host. In addition, we summarize research reporting the direct and indirect effects of the gut microbiota on NSAID disposition, efficacy, and toxicity, mainly related to lower GI side effects. Although we describe these interactions as separate events, in reality they are part of a dynamic and multidirectional interplay. Thus, NSAIDs may modify the composition of the intestinal microbiota and cause changes in the relative abundance of the bacterial strains involved in drug absorption and metabolism that ultimately affects NSAID therapeutic outcomes. Finally, we briefly highlight the translational implication of this research and discuss progress towards microbiota-based interventions to reduce NSAID induced lower GI side effects. In conclusion, the increasing understanding of the NSAID–gut microbiota interface could provide novel insights for the discovery and development of new anti-inflammatory drugs. Moreover, this knowledge could be used as a precision medicine-based approach to increase NSAID efficacy and prevent NSAID related toxicities.

## The Gut Microbiota

The gut microbiota is a large and diverse community of microbes that inhabit the GI tract. The microbiota interacts with human cells and these interactions are very diverse due to the variability of microbial organisms in the GI tract. The different regions of the gut vary in pH, oxygen levels, epithelial cell physiology, and nutrient content; therefore, the environment in the GI tract determinates the dominant bacterial strains in the different areas and the types of metabolic processes that occur (Koppel et al., 2017).

For example, the small intestine, characterized by higher oxygen levels, has a high proportion of facultative anaerobic bacteria, including *Lactobacillaceae*; whereas the colon and feces, characterized by lower oxygen levels, have a high proportion of strictly anaerobic bacteria, including *Bacteroidaceae, Prevotellaceae, Rikenellaceae, Lachnospiraceae*, and *Ruminococcaceae* (Gu et al., 2013).

The gut microbiota is a highly plastic community which is influenced by numerous factors like diet, gender, environment, eating behavior, and xenobiotic and microbial metabolites. It is constituted by bacteria, archaea, viruses, fungi, and parasites, counting approximately trillions of microorganisms. The number of microbes in the intestinal tract increases from the stomach to the colon, ranging approximately from 10^1^–10^4^ to 10^10^–10^12^ microbes per gram of luminal content, respectively (Sender et al., 2016). Bacteria are the most abundant components of the gut microbiota. There are over a thousand bacterial species in the gut, counting approximately the same order of bacterial cells as the number of human cells. Healthy human gut microbiota is diverse and largely consists of seven phyla: *Bacteroidetes*, *Firmicutes, Proteobacteria, Verrumicrobia, Actinobacteria*, *Fusobacteria, and Cyanobacteria* (Sommer and Bäckhed, 2013). Although obligate anaerobes typically dominate most intestinal anatomical locations, a large range of variation in community composition is observed among individuals and among locations within the gut (Eckburg et al., 2005; Huttenhower et al., 2012). Intestinal bacteria are involved in many human physiological processes: they metabolize structurally different food molecules, including lipid and glucose metabolism, and synthesize amino acids and vitamins. In addition, they play important roles in several physiological functions like intestinal epithelial barrier, GI sensory and motor activities, mucosal immunity, systemic immune surveillance, and host behavior (Thomas et al., 2017). The microbiome is estimated to comprise ~3.3 million microbial genes (150 times more genes than the host human genome), including genes involved in xenobiotics biodegradation and metabolic pathways (Qin et al., 2010; Spanogiannopoulos et al., 2016). The gut microbiota can be considered as an important metabolic “organ” for drugs, with a metabolic capacity at least equal to that of the liver.

The gut microbiota can directly cause chemical modifications of drugs themselves or of their metabolites. The human gut microbiota is known to biotransform so far more than 50 pharmaceuticals by producing enzymes with different catalytic activities and thus determining the bioactivity, bioavailability, and toxicity of several natural or synthetic substances (Koppel et al., 2017; Wilson and Nicholson, 2017; Collins and Patterson, 2020). Drugs taken orally can encounter gut microbes mainly in the small or large intestine or through biliary excretion.

The reactions catalyzed by bacterial enzymes include: reduction, hydrolysis, hydroxylation, dihydroxylation, dealkylation, deamination, decarboxylation, acetylation, deacetylation, and rarely oxidation (Sousa et al., 2008; Wilson and Nicholson, 2017). The major bacterial enzyme families involved in drug metabolism are: *β*-glucuronidases, azoreductases, demethylases, desulfatases, sulfoxide reductase, and cardiac glycoside reductases (Wilkinson et al., 2018; Collins and Pattersons, 2020). In contrast, the host enzymes, CYP P450 and phase II drug metabolizing enzymes, participate in drug metabolism mainly by oxidation or conjugation reactions (Sousa et al., 2008). Gut microbial metabolism of drugs generates metabolites with active, inactive, or toxic properties (Li et al., 2016). The formation of these microbial metabolites occurs concurrently and often competing with host metabolic processes. Thus, the chemistry of microbial transformations is distinct from that of host enzymes, and it can oppose or reverse host metabolism, ultimately altering the pharmacokinetics and pharmacodynamics of xenobiotics and their metabolites. In addition, whereas host metabolism occurs to detoxify the body from xenobiotics, microbial modifications occur generally to provide nutrients and energy to the microbes.

The synergism between the host and the microbiota generates metabolites that would not be synthesized by the host alone and can alter the biological activities and duration of xenobiotics. For example, the NSAID sulindac is a pro-drug that requires gut bacteria to be converted in the active compound sulindac sulfide (Strong et al., 1987). Additionally, bacterial metabolites can compete with drugs for host metabolic enzymes. For example, the production of p-cresol by bacteria competes with the human cytosolic sulfotransferase involved in the metabolism of acetaminophen, so that increase production of p-cresol causes decreased acetaminophen O-sulfonation and increased glucuronidation (Clayton et al., 2009). Another example is represented by the Parkinson’s disease medication Levodopa (L-dopa), which is metabolized first by a pyridoxal phosphate-dependent tyrosine decarboxylase from *Enterococcus faecalis*, followed by a molybdenum-dependent dehydroxylase from *Eggerthella lenta.* L-dopa degradation in human stool samples can be predicted predominantly by tyrosine decarboxylase gene expression and abundance of *Enterococcus faecalis* and *Eggerthella lenta* (Maini Rekdal et al., 2019). Moreover, bacterial enzymes, like *β*-glucuronidase, *β*-glucosidase, demethylase, desulfatases, and other phase II reversing enzymes, can remove small molecules attached to the drug by host enzymes during the drug metabolism. This process makes the free parent drug molecule available for reabsorption (enterohepatic circulation) by the host and thus increases the exposure of the host to the drug itself or its metabolites. This kind of reabsorption prolongs the exposure of drugs in the body (higher half-life) and often contributes to toxicity (Boelsterli et al., 2013).

In addition, the gut microbiota can indirectly influence the drug fate. The gut microbiota can limit drug absorption in the small intestine by increasing the expression of cell–cell adhesion proteins, thickening the protective mucosal layer, and/or directly sequestering chemicals to prevent their absorption (Collins and Pattersons, 2020). These processes can influence the bioavailability of the drugs with consequences for drug toxicity (at the body site where the drug is bioaccumulated) and/or drug effectiveness (since lower concentrations of drugs are circulating). Furthermore, the gut microbiota can regulate host expression of genes involved in vary metabolic pathways, including nuclear receptor regulation, phase I and II enzymes, and transporters (Collins and Pattersons, 2020). Moreover, the gut microbiota can produce microbial metabolites that can compete with drug metabolism (Sun et al., 2019).

Thus, microbes that reside in the human gut are considered a newly recognized modulator of drug exposure and consequently of variability in drug response, but they may also represent a potential source of new therapeutics.

## Impact of Nsaids on Gut Microbiota Composition and Metabolic Activity

Medication has recently emerged as one of the most influential determinants of the gut microbiota composition and activity (Falony et al., 2016; Zhernakova et al., 2016; Maier et al., 2018). Several classes of drugs can shape the physiome of the gut microbiota by shifting the composition of the intestinal microbial communities (Maurice et al., 2013; Mani et al., 2014). Some drugs determine a shift of the microbiota composition to favor the abundance of microbial taxa involved in its metabolism. This shift could consequently affect the pharmacokinetic proprieties of subsequent doses of the drug itself and the pharmacokinetics of co-administered medications (Walsh et al., 2018).

**Figure 1:**
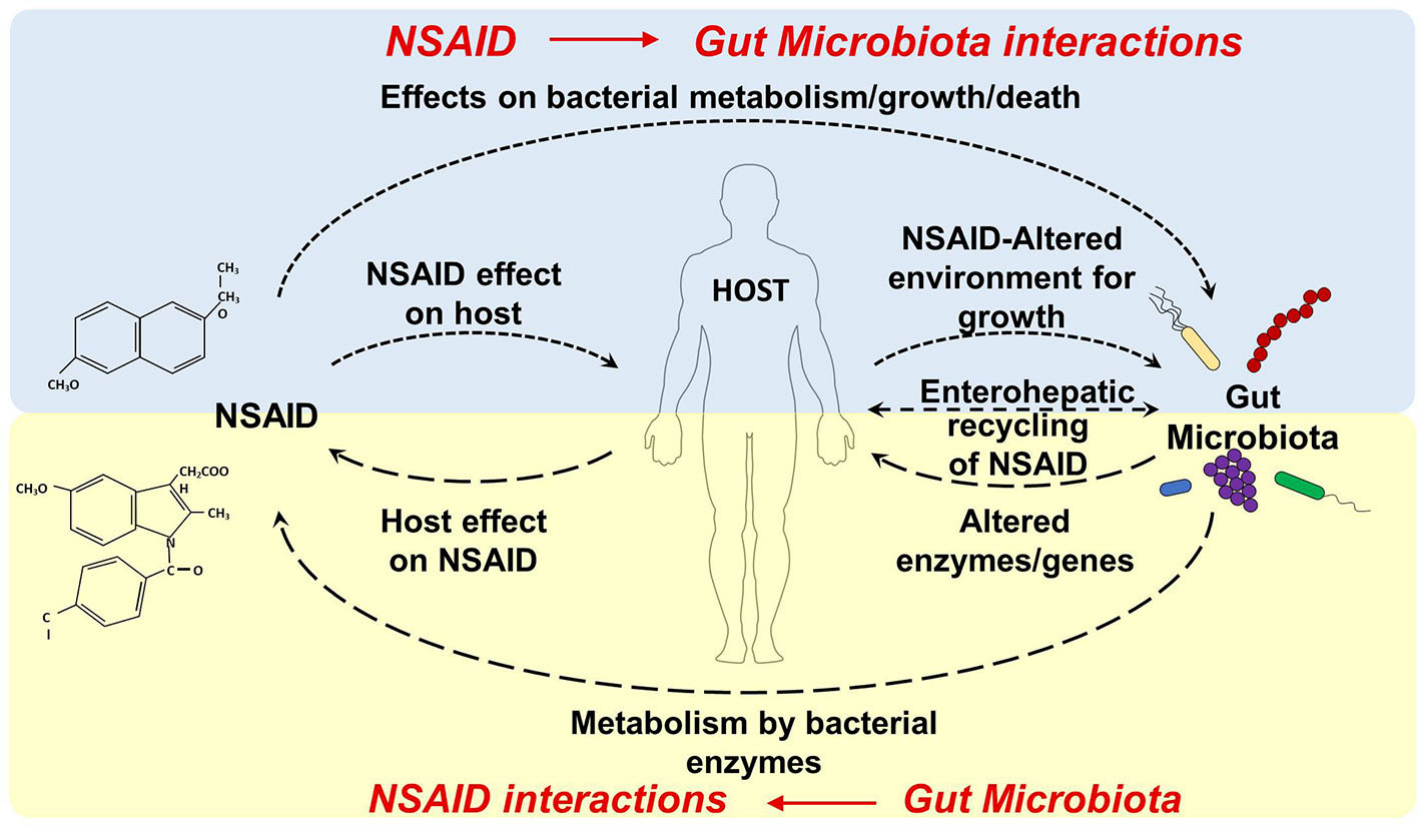
Dynamic interactions between NSAID and gut microbiota. NSAID Direct effects: NSAIDs can have antibacterial properties that directly affect the composition of the gut microbiota (by effecting bacterial metabolism/growth, and/or inducing microbial cell death). NSAID Indirect effect: NSAIDs can alter the physiological properties or functions of host organs (*e.g.* by changing the gut environment, changing mucosa integrity and permeability, and interfering with host and microbial metabolism) which may, in turn, precipitate in dysbiosis. Gut Microbiota Direct effects: the gut microbiota can directly biotransform orally and systemically administrated drugs into other chemical forms or metabolites, which may have altered efficacy or toxicity. Gut Microbiota Indirect effects: the gut microbiota can indirectly impact the efficacy and toxicity of drugs by altering the host metabolism capacity or processes (by influencing hepatic function, altering expression of hepatic enzymes or metabolic genes, interfering with detox pathway).

NSAID use can affect the gut microbiota composition and metabolic activity through a direct effect on the microbiota (*e.g.* by inhibiting/facilitating microbial growth, inducing microbial cell death and/or influencing microbial metabolism) or through an indirect effect by interacting with the host (*e.g.* by changing the metabolism, gut environment, mucosa integrity, and permeability, **Figure 1**). Both selective and nonselective NSAIDs can affect the composition of the gut microbiota in animals and in humans (**Table 2**).

**Table 2:** Studies assessing the perturbation of the gut microbiota composition by NSAIDs.

NSAID	Specie	Changes	Location	Reference
Indomethacin (s.c. 7.5 mg/kg)	Rat (females)	↑*Enterococcus faecalis* ↓ Segmented filamentous bacteria	Small intestine content; mesenteric lymph nodes	Dalby et al., 2006
Indomethacin (s.c. 7.5 mg/kg)	Rat (males)	↑ *Bacteroides* and *Enterobacteriaceae* ↑ *Clostridium*	Ileum and cecum-colon content	Terán-Ventura et al., 2014
Indomethacin (gavage, 10 mg/kg)	Mouse (males)	↑ *Firmicutes* ↑R*uminococcus*, ↑*Lachnospiraceae*, ↑*Anaeroplasma* ↑*rc4-4* ↓ *Bacteroides* ↓*S24-7*	Feces	Liang et al., 2015
Indomethacin (gavage, 10 mg/kg)	Mouse	↑*Firmicutes* ↓ *Bacteroides*	Feces	Xiao et al., 2017
Naproxen (gavage, 10 mg/kg BID)	Rat	*↓Lachnospiraceae* *↑Bacteroides*	Jejunum content	Syer et al., 2015
Diclofenac (gavage, 10 mg/kg BID)	Rat	*↑* Gram negative bacteria	Ileum	Reuter et al., 1997
Diclofenac (gavage, 4 mg/kg BID)	Rat	*↑ Proteobacteria* *↑Bacteroidetes* *↓ Firmicute*	Ileum	Colucci et al., 2018
Celecoxib (diet, 1,000 ppm)	APC ^Min/+^ mouse	*↑Coriobacteriaceae ↓Lactobacillaceae* and *↓Bifidobacteriaceae*	Ileal content and feces	Montrose et al., 2016
Rofecoxib (gavage, 5 mg/kg)	Rat	None	Jejunal content	Lázár et al., 2019
Aspirin (gavage, 0.23 g/kg)	Rat	*↑ Lactobacillus*	Oral swap	Cheng et al., 2018
Aspirin (gavage, 100 mg/kg)	Rat	No effect on enteric aerobic bacteria count	Ileum	Reuter et al., 1997
Celecoxib (os, 200 mg, BID)	Human (obese women)	None	Feces	Bokulich et al., 2016
Indomethacin (os, 75 mg, BID)	Human (males and females)	*↑Bacteroidetes* (♀) *↑Prevotellaceae* (♀) *↑Bacteroidetes* (♀) *↓Firmicutes* (♀) *↑Firmicutes* (♂) *↓ Proteobacteria* (♂) *↓ Alphaproteobacteria* (♂) *↓ Proteobacteria* (♂) *↓ Rhizobiales* (♂) *↓ Proteobacteria* (♂) *↓Pseudomonadaceae* (♂)	Feces Duodenal aspirates	Edogawa et al., 2018

### Animal Studies

Several animal studies have shown that NSAID administration causes significant changes in the intestinal microbiota, often increasing the abundance of Gram-negative bacteria (Kent et al., 1969; Honda et al., 1999; Otani et al., 2017; Vázquez-Baeza et al., 2018). Subcutaneous treatment with indomethacin increases intestinal *Enterococcus faecalis* and decreases segmented filamentous bacteria in the small intestine and mesenteric lymph nodes in female rats (Dalby et al., 2006). Similarly, systemic administration of indomethacin causes intestinal inflammation, characterized by upregulation of Toll-like receptor (TLR)-2 and -4 in the intestine and increases the abundance of *Bacteroides* and *Enterobacteriaceae* in the ileum and cecum-colon and *Clostridium* the in ileum of male rats (Terán-Ventura et al., 2014). In mice, a single oral administration of indomethacin causes lesions in the small intestine, increases the richness of the microbiota in the large intestinal content and feces, with an expansion of pro-inflammatory bacteria, reduces the diversity of the microbial species in the cecum and large intestinal mucosal content, and increases the microbial diversity in the feces. Meanwhile, one-week treatment with a diet containing indomethacin decreases the diversity of the microbiota only in the cecum luminal content, probably indicating a recovery of the gut microbiota during chronic drug exposure (Liang et al., 2015). In contrast, another study reports that a single oral administration of indomethacin does not cause small intestinal damage but induces adaptive beneficial changes in the gut microbiota, including increased abundance of *Firmicutes* and decreased abundance of *Bacteroidetes*, which may be protective against indomethacin-induced enteropathy in mice (Xiao et al., 2017). In fact, the administration of antibiotics after indomethacin treatment increases the mortality due to GI lesions, while naïve mice transplanted with adaptively changed microbiota collected from mice previously treated with indomethacin, show smaller intestinal damage in response to indomethacin.

Diet and dietary additives can increase the vulnerability to indomethacin induced-enteropathy. High fat diet aggravates indomethacin-induced small intestinal damage in mice *via* intestinal dysbiosis, increases gene expression of interleukin (IL)-17A in the intestine and augments intestinal permeability (Sugimura et al., 2019). A dietary emulsifier, polysorbate 80, exacerbates indomethacin-induced small-intestinal lesions *via* intestinal dysbiosis and increases protein expression of IL-1*β* in the small intestine (Furuhashi et al., 2019). A possible mechanism by which indomethacin can cause the translocation of luminal bacteria from the lumen into the mucosa is by altering the content of sugars and lipids in the glycocalyx layer of the mucosa (Basivireddy et al., 2005). Indomethacin-induced dysbiosis has also been associated with intestinal infections. Indomethacin increases the severity of *Clostridium difficile* infection (CDI) by perturbing the gut microbiota and dysregulating intestinal inflammatory response in a mouse model of antibiotic-associated CDI (Maseda et al., 2019). Similarly, both low and high doses of indomethacin increase the severity of *Clostridium difficile* infection in two different mouse models of antibiotic-associated CDI (Muñoz-Miralles et al., 2018). Such findings support epidemiological data linking NSAID exposure and CDI and warn against the use of NSAIDs in patients at high risk for *Clostridium difficile* (Permpalung et al., 2016).

Short oral treatment with naproxen causes intestinal ulceration and inflammation, increases bile cytotoxicity, and shifts the jejunal microbiota composition in rats (Syer et al., 2015). Naproxen decreases the abundance of *Lachnospiraceae* family (from the Gram-positive *Clostridia* class) and increases the *Bacteroides* genus (from the Gram-negative *Bacteroidia* class) in the rat jejunum (Syer et al., 2015). Similarly, short term oral administration of diclofenac increases intestinal permeability and ulceration and augments the number of Gram-negative bacteria in rat ileum (Reuter et al., 1997). In a recent study, intragastrical treatment with diclofenac for 14 days caused small intestinal damage and inflammation characterized by an increased protein expression of TLR-2 and TLR-4 and biosynthesis of IL-1*β* and tumor necrosis factor-*α* (TNF-*α*) (Colucci et al., 2018) in the ileum. Moreover, diclofenac-induced enteropathy is associated with an increase in the relative abundance of *Proteobacteria* and *Bacteroidetes* and a decrease in *Firmicutes* in the rat ileum.

Chronic oral treatment with celecoxib also alters the intestinal microbiota, causing an increase in *Coriobacteriaceae* and decrease in *Bifidobacteriaceae* and *Lactobacillaceae* abundance in both ileal content and feces, and this effect is associated with the reduction of polyp burden in APC ^Min/+^ mice. Moreover, celecoxib alters the fecal metabolite profile and diminishes the content of amino acids, dipeptides, lipids, nucleotides and glucose (Montrose et al., 2016). In contrast, a long-term intragastrical treatment with rofecoxib does not cause small intestinal damage and does not influence the composition, richness, and diversity of the jejunal microbiota in rats (Lázár et al., 2019). The result of this study may indicate that, in addition to COX-2 inhibition, the microbial alterations caused by some NSAIDs may be due to drug-specific properties that determine topical epithelial damage.

There is also some initial evidence that NSAIDs can alter the oral microbial composition. Two-weeks of intragastrical treatment with aspirin increases oral microbial diversity and content in rats (Cheng et al., 2018). In particular, the *Lactobacillaceae* abundance is increased significantly by aspirin. Furthermore, aspirin reduces the immunoglobulin (Ig)G and secretory IgA content in the saliva. On the contrary, a short oral treatment with aspirin does not have an impact on the Gram-negative bacterial counts in the ileum and does not cause small intestinal damage in rats (Reuter et al., 1997).

Although the overgrowth of specific bacteria after NSAID administration has been recognized for several decades, the reasons for NSAID-induced dysbiosis are not known. One hypothesis is that certain NSAIDs could have antibacterial activity as reported *in vitro* for indomethacin, diclofenac, ibuprofen, aspirin and celecoxib (Annadurai et al., 2002; Shirin et al., 2006; Obad et al., 2015; Thangamani et al., 2015; Chan et al., 2017; Maier et al., 2018). In mouse infection models, diclofenac has shown to be effective against Salmonella Typhimurium (Dastidar et al., 2000; Annadurai et al., 2002), and celecoxib has shown to be efficacious in a methicillin-resistant *S. aureus* skin infection (Thangamani et al., 2015). However, it still remains unknown whether the antibacterial activity of NSAIDs occurs with therapeutic concentrations of the drugs *in vivo*; it could be responsible for the dysbiosis observed with NSAIDs, and it could be implicated in NSAID-induced enteropathy.

### Human Studies

The impact of NSAID administration on the human intestinal microbiota has been less explored due to the difficulties associated with this type of investigations. NSAID users can present different gut microbiota profiles from that of nonusers, as do users of specific types of NSAIDs (Rogers and Aronoff, 2016). For example, treatment with aspirin causes a shift in the composition of the gut microbiota regarding *Prevotella, Bacteroides*, *Ruminococcaceae*, and *Barnesiella*, whereas celecoxib and ibuprofen increase the abundance of *Acidaminococcaceae* and *Enterobacteriaceae*. Ibuprofen causes enrichment in *Propionibacteriaceae*, *Pseudomonadaceae*, *Puniceicoccaceae*, *and Rikenellaceae* species compared with either nonusers or naproxen users.

The composition of the gut microbiota of NSAID and PPI users differs from that of only NSAID users in the abundance of *Bacteroides* and *Erysipelotrichaceae* species. Furthermore, *Bacteroides* species and a bacterium of family *Ruminococcaceae* differ between NSAID users and antidepressant and laxative users (Rogers and Aronoff, 2016). Thus, these data indicate that the profile of bacteria in the GI tract reflects the combinations of medicines ingested. This is in agreement with the fact that the co-administration of drugs may cause changes in the composition of the microbiota to favor the abundance of taxa that have metabolizing capacity for those drugs (Ticinesi et al., 2017). In contrast to the aforementioned study, celecoxib does not alter the composition of the gut microbiota in a longitudinal study in post-menopausal obese women (Bokulich et al., 2016). In this small study, the inter-individual variability in response to celecoxib could have masked the effect of this drug on the diversity of the gut microbiota. On this note, a recent study reported that without altering the composition of the microbiota, celecoxib can decrease microbial butyrate production in a human intestinal microbiota ecosystem model and also diminish markers of inflammation like IL-8 and CXCL16 in intestinal cells *in vitro* (Hernandez-Sanabria et al., 2020).

Although these are the first studies investigating a possible shift in the composition of the gut microbiota by NSAIDs in humans, some of these studies did not take into account confounding factors (*e.g.* primary disease conditions, concomitant use of other medications, drug dose, drug exposure, time of drug intake, sex, diet, and age) and lacked appropriate controls. Indeed, both age and sex can influence the impact of NSAIDs on the composition of human gut microbiota. In terms of age-related differences, the total number of microbes is reduced in older (between 70 and 85-year-old) compared with younger subjects (mean age 28 years), but it is higher in the senior NSAID users compared with senior nonusers. However, the fecal microbiota composition of older subjects using NSAIDs presents a reduction of the abundance in *Collinsella*, *Actinobacteria*, and *Lactobacilli*, compared to older nonusers and young adults (Mäkivuokko et al., 2010).

In terms of sex-related differences, women present lower intestinal permeability and higher microbial diversity than man (Edogawa et al., 2018). Intestinal permeability is sensitive to perturbation following acute treatment with indomethacin, but returns to baseline 4–6 weeks after discontinuation of the drug in both sexes. Fecal and duodenal gut microbiota diversity decreases after acute treatment with indomethacin, especially in women, which recovers 4–6 weeks later. Women present greater abundance of *Actinobacteria* phylum but lower abundance of *Bacteroidetes* and *Proteobacteria* compared to men (Edogawa et al., 2018).

In addition to the demographic factors, psychological stress can exacerbate indomethacin-induced small bowel injury in mice by increasing the total number of bacteria and the proportion of gram-negative bacteria and by increasing the permeability of intestinal mucosa *via* glucocorticoid receptor signaling (Yoshikawa et al., 2017). This study indicates that the microbiota–gut–brain axis may play an important role in the development of NSAID-induced enteropathy as reported also for other pathological conditions (Collins and Bercik, 2009).

Future studies should be designed to include larger and more diverse cohorts and be carried longitudinally. Moreover, more work and complex analysis (*e.g.*, multi-omics and longitudinal studies) are needed to go beyond associations and determine causality.

## Impact of the Gut Microbiota on Nsaid Disposition, Toxicity and Efficacy

The gut microbiota can have direct and indirect effects on drug absorption, distribution, metabolism and excretion, and consequently affect drug efficacy and toxicity (Wilson and Nicholson, 2017). In the case of the NSAIDs, the gut microbiota can directly biotransform orally and systemically administrated drugs into other chemical forms or metabolites, which may have altered efficacy or toxicity (**Figure 1**). Different microbial, fungal, and yeast cultures are capable of biotransforming valdecoxib and celecoxib *in vitro* (Keshetti and Ciddi, 2010; Srisailam and Veeresham, 2010). Moreover, flurbiprofen is converted by the zygomycete fungus *Cunninghamella* to a variety of metabolites in different mammals, including humans (Amadio et al., 2010). Furthermore, the gut microbiota can indirectly impact the efficacy and toxicity of drugs by altering the host metabolism capacity (by influencing hepatic function, altering expression of hepatic enzymes or metabolic genes, interfering with detox pathway) (**Figure 1**).

While few studies show the influence of the gut microbiota on NSAID metabolism and efficacy, several reports indicate its involvement in NSAID-induced lower GI toxicity. Compositional changes of the gut microbiota associated with indomethacin administration can alter both its disposition and consequently its inhibitory effect on prostanoid biosynthesis *via* de-glucuronidation of its metabolites during enterohepatic recirculation. Antibiotic suppression of intestinal bacteria significantly reduces the level of indomethacin de-glucuronidation, resulting in increased elimination, a shortened half-life, and reduced drug exposure (Liang et al., 2015).

Reduction of metabolic activity of the gut microbiota by oral antibiotics reduces the host metabolism of orally administrated aspirin and increases its antithrombotic effects in rats. However, aspirin metabolism is not changed by antibiotics administrated intravenously (Kim et al., 2016). This study represents the first evidence of the impact of the gut microbiota on the NSAID effects on the CV system. Similarly, the oral administration of amoxicillin reduces the pharmacokinetics of aspirin by slowing down the metabolic activity of the gut microbiota involved in the biotransformation of aspirin in rats (Zhang et al., 2019). These findings indicate that antibiotics could interfere with the gut microbiota metabolism of some NSAIDs, frequently prescribed together in the clinical setting, and affect their bioavailability and efficacy.

## NSAID-Induced Enteropathy

The mechanisms underlying NSAID-induced enteropathy are still not completely understood. Our current knowledge indicates that the pathogenesis of NSAID-induced small intestinal damage is a multifactorial process that occurs in response to multiple insults (Davies et al., 2000; Boelsterli et al., 2013). NSAIDs can cause intestinal damage through topical irritant effects due to the direct contact of the drug with the intestinal mucosa, local inhibition of protective PGs, and interaction with the gut microbiota.

The topical effects are caused by physiochemical proprieties of the drugs. These are COX-independent effects linked to the drug acidity and/or lipophilicity (most NSAIDs are lipid-soluble weak organic acids). The topical effects caused by NSAIDs are relevant to oral drug administration but also to parental administration due to hepatobiliary excretion of active metabolites and their enterohepatic cycling (Boelsterli et al., 2013). On the contrary, the local inhibition of protective PGs is a COX-dependent effect related to the drug potency and selectivity for the inhibition of COX isozymes. Indeed, there are differences between individual NSAIDs and their risk of inducing small intestinal damage in humans (Sigthorsson et al., 1998; Davies et al., 2000).

Drugs present in the intestinal lumen initially interact with the intestinal mucus layer and the cell surface phospholipid bilayer, where they can trigger drug-induced mithocondrial and endoplasmic reticulum damage, and oxidative stress in epithelial cells, all detrimental events that coalesce to impair healthy GI function. These topical effects strongly increase the permeability of the intestinal mucosa and lead to a low-grade inflammation, which facilitates the entrance and action of luminal aggressors (bile, intestinal enzymes, and commensal bacteria) in the small bowel (Bjarnason et al., 2018). The concurrent COX inhibition and the presence of luminal aggressors increase the severity of inflammatory and ulcerative damage causing mucosal erosions and ulcers (Bjarnason et al., 1993; Somasundaram et al., 1997; Wallace, 2012). Indeed, NSAID administration can increase intestinal permeability within 12–24 h after drug intake, mainly in the jejunum and ileum, and cause inflammation in the small intestine within 10 days, while ulcer formation can take up to 2 weeks (Bjarnason et al., 2018).

There are controversial pieces of evidence regarding which COX isozymes are expressed in the intestine and the degree of their involvement in the development of lower GI toxicity. In the small intestine, PGs are implicated in the maintenance of blood flow, turnover of epithelial cells, mucus secretion, intestinal motility, mucosal repair, and inflammatory response. Intestinal PGs are produced mainly by COX-1 since COX-2 seems to have little or no gene/protein expression in the healthy intestine (Kargman et al., 1996; Takeuchi et al., 2010a; Bjarnason et al., 2018). However, some reports indicate constitutive expression of COX-2 gene/protein in the intestine of some animal species (Haworth et al., 2005; Fornai et al., 2014; Kirkby et al., 2016). Additional studies indicate that COX-1 is mainly responsible for the production of endogenous PGs involved in mucosal protection, with COX-2 contributing to mucosal defense only under conditions or inflammation, while, while both COX-1 and COX-2 are involved in the healing of small intestinal lesions (Takeuchi and Amagase, 2018).

Genetic and pharmacological studies in animal models have helped to understand the pathophysiological functions of COX-1 and COX-2 and of their products in the GI tract. Cox-1 deficient mice do not exhibit spontaneous gastric ulcers despite low mucosal PG levels (Langenbach et al., 1995; Loftin et al., 2002). The gastric pH is lower in Cox-1 deficient mice than in wild-type and Cox-2 deficient mice (Langenbach et al., 1999), consistent with the Cox-1 dependent secretion of acid and/or bicarbonate in the stomach. Thus, although the suppression of COX-1-derived PGs increases stomach acidity, this is not sufficient to induce gastric lesions. Traditional Cox-2 deficient mice, which have a reduced life-span due cardio-renal defects, present normal PG level and no gastric ulcers (Morham et al., 1995; Loftin et al., 2002). Similarly, rodents treated with the COX-1 inhibitor SC-560 or with selective COX-2 inhibitors do not present upper GI lesions (Futaki et al., 1993; Masferrer et al., 1994; Wallace et al., 2000; Gretzer et al., 2001; Tanaka et al., 2001). Indeed, dual inhibition of both COX-1 and COX-2 is needed to cause damage in the upper GI tract as observed with nonselective NSAIDs and with COX-1 or COX-2 inhibitors in rodents (Langenbach et al., 1995; Wallace et al., 2000; Tanaka et al., 2001).

Similarly, in the small intestine, Cox-1 deficiency or inhibition with SC-560 does not cause ulcers in mice despite a significant reduction in intestinal PGE_2_ levels (Sigthorsson et al., 2002), while Cox-2 deficient mice present peritonitis with intestinal inflammation and fibrosis (Morham et al., 1995). Short term COX-2 inhibition with celecoxib or rofecoxib does not reduce intestinal mucosal PGE_2_ and does not cause intestinal damage (Sigthorsson et al., 2002; Tanaka et al., 2002a; Ohno et al., 2004; Hotz-Behofsits et al., 2010), while long-term Cox-2 deficiency or inhibition leads to disruption of the small bowel barrier function, intestinal chronic inflammation, and microscopically ileal ulcers in male and female mice despite normal PGE_2_ levels (Sigthorsson et al., 2002). However, the lesions caused by long-term COX-2 suppression have a different localization (terminal ileum *vs* mid to small intestine) and histopathologic appearance compared to the ones caused by acute exposure to nonselective NSAIDs (Sigthorsson et al., 2002). In contrast, long-term COX-2 inhibition with rofecoxib does not reduce intestinal mucosal PGE_2_ content, does not cause mucosal damage, and does not alter the composition of the intestinal microbiota in male rats (Lázár et al., 2019). The results of these studies indicate that other factors (*i.e.* sex, age, diet, environment, drug metabolism, *physicochemical properties of* the drug), in addition to COX-2 inhibition, maybe be contributing to the formation of the intestinal damage observed with long term exposure to celecoxib.

Dual inhibition of COX isozymes (with nonacidic compounds like SC-560 and rofecoxib or celecoxib) causes lower GI damage as well the use of COX-1 or COX-2 inhibitors in Cox-2 or Cox-1 deficient mice (Sigthorsson et al., 2002; Tanaka et al., 2002a; Hotz-Behofsits et al., 2010; Takeuchi et al., 2010a; Takeuchi and Satoh, 2015). Similarly, NSAIDs that inhibit both COXs, such as indomethacin, diclofenac, naproxen, and flurbiprofen, severely damage the small intestine in rodents (Reuter et al., 1997; Konaka et al., 1999; Tanaka et al., 2002a; Syer et al., 2015; Takeuchi and Satoh, 2015; Colucci, et al., 2018).

These studies indicate that inhibition of both COX isozymes is required for the development of intestinal lesions after short NSAID exposure, with or without the presence of an NSAID that causes topical effects (Sigthorsson et al., 2002; Hotz-Behofsits et al., 2010). Intestinal damage can also result as a consequence of COX-2 deletion or inhibition and the presence of an NSAID with topical effects as shown in Cox-2 deficient mice treated with (R)-2-phenyl propionic acid, a compound that causes topical irritant effects but does not have COX inhibitory activity, and in wild-type mice treated with celecoxib and (R)-2-phenyl propionic acid (Hotz-Behofsits et al., 2010). COX-1 inhibition alone is not sufficient to cause lower GI damage probably because COX-1 inhibition triggers compensatory mechanisms to overcome the functional consequences of PG deficiency like upregulation of COX-2 or inducible nitric oxide gene/protein expression in the intestinal mucosa (Tanaka et al., 2002a; Tanaka et al., 2002b; Takeuchi et al., 2010a; Takeuchi and Satoh, 2015). Moreover, these studies indicate that suppression of prostaglandin synthesis by NSAIDs seems unlikely to be a major contributor to the development of NSAID-induced small intestinal injuries compared to other factors since both Cox-1 deficient mice and wild-type mice treated with SC-560 do not present intestinal damage, although they present a significant reduction of intestinal PGE_2_ (Melarange et al., 1992; Reuter et al., 1997; Sigthorsson et al., 2002).

Many of the results obtained in animal studies were confirmed by clinical trials with coxibs. These drugs present equal clinical efficacy compared to nonselective NSAIDs and improved gastric tolerability, as assessed by short-term (Lanza et al., 1999) or long-term (Simon et al., 1999; Hawkey et al., 2000) endoscopic studies and by GI outcomes studies (Bombardier et al., 2000; Silverstein et al., 2000). On the contrary, long exposures with coxibs and nonselective NSAIDs cause similar prevalence of small bowel damage, indicating a role for COX-2 in maintaining the mucosal integrity in the small intestine (Maiden et al., 2007; Maiden, 2009). However, selective COX-2 inhibitors may cause less severe mucosal lesions than nonselective NSAIDs (Maehata et al., 2012).

Although Cox-1 or Cox-2 deficient mice do not present spontaneous intestinal ulcers, Cox-1 deficient mice and mice treated with a COX-1 or a COX-2 inhibitor retarded healing of pre-existing ulcers (Blikslager et al., 2002; Hatazawa et al., 2006). It is noteworthy to mention that of the healing process, PGE_2_ is produced mainly by COX-2, while in the late phase PGE_2_ is produced mainly by COX-1 activity (Hatazawa et al., 2006; Takeuchi and Amagase, 2018).

The effect of PGE_2_ in mediating the healing of small bowel lesions occurs mainly through PGE_2_ receptor 4 (EP4), and this effect is functionally associated with stimulation of angiogenesis *via* the up-regulation of vascular endothelial growth factor expression (Takeuchi et al., 2010b; Takeuchi, 2014). Indeed, supplementation with PGE_2_ analogs prevents NSAID-induced enteropathy and promotes the healing of intestinal ulcers in animal models (Kunikata et al., 2001; Kunikata et al., 2002; Hatazawa et al., 2006), however, the FDA-approved oral PGE_1_ analogue misoprostol showed mixed results in clinical studies (Bjarnason et al., 1989; Davies et al., 1993; Watanabe et al., 2008b; Fujimori et al., 2009; Kyaw et al., 2018; Taha et al., 2018). In addition, misoprostol has often shown unfavorable side effects such as diarrhea, abdominal pain, or bloating, and therefore, it is not generally suitable for its long-term use (Handa et al., 2014).

The abundance and the diversity of bacteria present in the small intestine play an important role in the pathogenesis of NSAID-induced enteropathy. NSAID use can modify the composition of the gut microbiota and induce mainly the overgrowth of Gram-negative and anaerobic bacterial species, which, possibly through release of endotoxin or microbial metabolites, lower mucosal defense and increase the susceptibility to intestinal damage (Hagiwara et al., 2004; Lanas and Scarpignato, 2006; Scarpignato, 2008; Syer et al., 2015). Germ-free rodents develop little or no intestinal lesions after NSAID exposure, but when colonized by specific Gram-negative or Gram-positive bacteria, these animals become sensitive to NSAID-induced small intestinal damage. For example, germ-free rats are resistant to indomethacin-induced enteropathy, in contrast germ-free rats colonized with *Escherichia coli* develop severe lesions in the gut (Robert and Asano, 1977). Moreover, a COX inhibitor, 5-bromo-2-(4-fluorophenyl)-3-(4-methylsulfonylphenyl) thiophene (BFMeT), is unable to induce ulcers in germ-free rats and in gnotobiotic rats mono-associated with *Lactobacillus acidophilus* or *Bifidobacterium adolescentis;* whereas BFMeT induces ileal ulcers in gnotobiotic rats colonized with *Eubacterium limosum* or *Escherichia coli* (Uejima et al., 1996). Several studies (**Table 3**) have reported that the treatment with large spectrum antibiotics can reduce the severity of NSAID-induced intestinal damage in animal models (Kent et al., 1969; Uejima et al., 1996; Koga et al., 1999; Leite et al., 2001) and in humans (Bjarnason et al., 1992; Scarpignato et al., 2017). For example, indomethacin-induced intestinal damage is partially prevented by the pre-treatment with poorly absorbed antibiotics in rats (Konaka et al., 1999; Fornai et al., 2016). Also, naproxen causes a significant shift in the microbiota composition of rats, and treatment with a cocktail of antibiotics reduces the severity of naproxen-induced small intestinal ulceration (Syer et al., 2015). Diclofenac-induced enteropathy is reduced by rifaximin, a broad-spectrum oral antibiotic, through both anti-bacterial and anti-inflammatory activities in rats (Colucci et al., 2018). In addition, some studies propose that antibiotic treatment may also facilitate the healing of intestinal lesions (Kent et al., 1969; Zwolinska-Wcislo et al., 2011). In addition, metronidazole, an antimicrobial targeting most Gram-negative and Gram-positive anaerobic bacteria, reduces the occurrence of NSAID-induced enteropathy in rats and in humans (Bjarnason et al., 1992; Yamada et al., 1993). However, the fact that antibiotics cannot completely prevent the NSAID-induced ulceration indicates that additional factors are involved in causing the initial intestinal damage.

**Table 3:** *In vivo* studies reporting the impact of antibiotic treatment on NSAID disposition, toxicity and efficacy.

NSAID	Antibiotic treatment	Specie	Changes on NSAID disposition, toxicity and efficacy	Reference
5-bromo-2‐(4‐fluorophenyl)‐3‐(4‐methylsulfonylphenyl) thiophene (BFMeT, 500–1,500 mg/kg, p.o.)	bacitracin, neomycin, streptomycin (200 mg/kg)	Rat	↓ ileal ulcers	Uejima et al., 1996
Aspirin (5 mg/kg, p.o.)	ampicillin (250 mg/kg)	Rat	*↑* absorption *↑* Cmax *↑* bleeding time	Kim et al., 2016
Aspirin (37.5 mg/kg, p.o.)	amoxicillin (157.5 mg/kg)	Rat	*↑* absorption *↑* Cmax	Zhang et al., 2019
Diclofenac (4 mg/kg, BID, p.o.)	rifaximin (50 mg/kg, BID, p.o.)	Aged Rat (males)	*↓* length of ileum lesions *↓* intestinal inflammation *↓* intestinal permeability *↑* hemoglobin	Colucci et al., 2018
Indomethacin (7.5 mg/kg, s.c.)	metronidazole (100 mg/kg, i.g.)	Rat (males)	*↓* intestinal permeability *↓* bacterial translocation to mesenteric lymph nods	Yamada et al., 1993
Indomethacin (5–40 mg/kg, p.o. or i.m.)	neomycin sulfate (1.3 g/L), polymvxin B sulfate (60 mg/L), bacitracin (30,000 U./L)	Rat	*↓* ulcer severity in the distal jejunum and the ileum	Kent et al., 1969
Indomethacin (10 mg/kg, s.c.)	ampicillin (800 mg/kg, p.o.)	Rat	↓ n. of enterobacteria in the mucosa ↓ occurrence of intestinal lesions	Konaka et al., 1999
Indomethacin (24 mg/kg, rectal injection)	kanamycin sulfate (1, 10, 100 mg/day)	Rat	*↓* Incidence of small intestinal ulcers	Koga et al., 1999
Indomethacin (7.5 mg/kg, p.o.)	metronidazole (60 mg/kg/dose)	Rat	*↓* intestinal damage *↓* intestinal permeability	Leite et al., 2001
Indomethacin (10 mg/kg, p.o.)	neomycin (1 g/L), vancomycin (0.5 g/L)	Mouse (males)	*↓* drug elimination *↓* half-life *↓* drug plasma concentration *↓* inhibition prostanoids	Liang et al., 2015
Indomethacin, (1.5 mg/kg BID)	rifaximin (50 mg/kg BID, i.g.)	Rat	↓ Intestinal lesions in the jejunum and in the ileum ↓ Intestinal inflammation and tissue peroxidation	Fornai et al., 2016
Indomethacin (10 mg/kg, p.o.)	neomycin (250 mg/kg), polymycin (9 mg/kg), metronidazole (50 mg/kg)	Mouse	*↑* mortality	Xiao et al., 2017
Naproxen (20 mg/kg, p.o.)	ampillicin (1 g/L), vancomycin (500 mg/L), neomycin (1 g/L), metronidazole (1 g/L)	Rat	*↓* Intestinal damage score	Syer et al., 2015
NSAIDs	metronidazole (800 mg/day, p.o.)	Human	*↓* intestinal inflammation *↓* blood loss	Bjarnason et al., 1992
Diclofenac (75 mg) + omeprazole (20 mg)	rifaximin (400 mg, p.o.)	Human	↓ proportion of subjects developing at least 1 small-bowel mucosal break	Scarpignato et al., 2017

The use of other drugs co-prescribed with NSAIDs, like for example PPIs, can have deleterious effects on small-bowel lesions, possibly through a combination of intestinal dysbiosis and increased intestinal permeability. In rats, PPIs significantly exacerbate naproxen- and celecoxib-induced intestinal ulceration and bleeding by causing a reduction of the jejunal content of *Actinobacteria* and *Bifidobacteria*, probably through changes of the pH in the GI tract over an extended period of time (Wallace et al., 2011). In germ free mice, the colonization with *Bifidobacteria*-enriched intestinal flora prevents the NSAID and PPI-induced small intestinal damage, whereas the colonization with bacteria from PPI-treated rats facilitates the development of NSAID-induced enteropathy (Wallace et al., 2011). Similarly, a recent study reports that PPIs aggravates indomethacin induced-enteropathy by reducing the population of *Lactobacillus Johnsonii* in the small intestine of mice (Nadatani et al., 2019). Consistent with the results of these animal studies, human data revealed that PPI use represents a risk factor for NSAID-induced small intestinal damage (Watanabe et al., 2013; Endo et al., 2014; Lué and Lanas, 2016; Washio et al., 2016). In addition, a meta-analysis of clinical studies comparing small intestinal bacterial overgrowth (SIBO) risk among adult users of PPIs *vs* nonusers indicates that the use of PPIs is associated with SIBO, a condition that can cause excessive fermentation and inflammation, leading to a variety of clinical complaints including bloating and diarrhea (Lo and Chan, 2013). Thus, dysbiosis secondary to PPI use may exacerbate the NSAID-enteropathy.

The involvement of Gram-negative bacteria in the pathogenesis of NSAID-induced enteropathy seems to be linked to the activation of toll like receptor (TLR)4 that enhances inflammation and contributes to intestinal lesions (Watanabe et al., 2008a; Nadatani et al., 2012; Higashimori et al., 2016). Increased intestinal permeability, migration of bacteria through the epithelium into the deeper layers of the mucosa, and mucosal inflammatory and immune response can be observed when the mucosal barrier function is disrupted by NSAID-mediated topical effects and prostanoid inhibition. Lipopolysaccharide (LPS) and high mobility group box 1 (HMGB1), when present in the lumen, can activate NLRP3 inflammasome through the binding to TLR4 in the intestinal cells, causing infiltration of neutrophils and macrophages and resulting in deep ulceration of the small intestinal mucosa. The activation of macrophages causes the release of pro-inflammatory cytokines such as TNF-*α*, IL-1*β*, neutrophil recruiting chemokines, and nitric oxide, which then induce neutrophil infiltration into the intestinal mucosa and submucosa. Neutrophil activation damages the small intestine through the release of cytotoxic agents like reactive oxygen species, elastases, and proteases (Bertrand et al., 1998; Konaka et al., 1999; Whittle, 2003; Hagiwara et al., 2004; Watanabe et al., 2008a; Nadatani et al., 2012; Whitfield-Cargile et al., 2016). A recent study also indicates a role for Gram-positive microorganisms in the pathogenesis of NSAID-induced enteropathy since increased protein expression of both TLR2 (which binds the outer membrane of Gram-positive bacteria) and TLR4 (which binds major cell wall components of Gram-negative bacteria) and an increased biosynthesis of IL-1*β* have been reported in a rat model of diclofenac-induced small intestinal damage (Colucci et al., 2018).

Neutrophils are important effector cells involved in NSAID-induced small intestinal damage since depletion of neutrophils from mice or rats reduced intestinal lesion formation in response to NSAIDs (Chmaisse et al., 1994; Watanabe et al., 2008a). On the other hand, macrophages that reside in the small intestine regulate the integrity of the epithelial barrier *via* secretion of IL-10 (Morhardt et al., 2019). This anti-inflammatory cytokine plays a critical role in intestinal homeostasis and in the restoration of the epithelial barrier after NSAID-driven damage, in a process that does not seem to be directly regulated by T and B cells or the gut microbiota (Morhardt et al., 2019). T cells seem dispensable to trigger NSAID-induced enteropathy since both euthymic and athymic nude rats develop intestinal ulcers following administration of indomethacin to the same degree than conventional rats (Koga et al., 1999). However, deficiency in specific T-cell subsets like, *γδ* T-cells in intestinal intraepithelial lymphocytes (IEL), leads to an increased sensitivity to NSAID-induced small intestinal damage in mice (Sumida et al., 2017). Additionally, GPR55-deficiency or antagonism protects mice from indomethacin-induced intestinal leakage since GPR55 acts as a negative regulator of *γδ* T-cell IEL accumulation and homing to the small intestine, and it also antagonizes indomethacin-induced *γδ* T-cell egress from Peyer’s patches (Sumida et al., 2017). Moreover, Peyer’s patches play protective roles in indomethacin-induced enteropathy through the induction of immune-regulatory CD103^+^ dendritic cells and IL-10-expressing CD4^+^ T cells in the gut-associated lymphoid tissue (Hiyama et al., 2014).

Most NSAIDs, including derivatives of enolic acid (piroxicam), propionic acid, (such as ketoprofen, naproxen, ibuprofen and flurbiprofen), and acetic acid (such as diclofenac and indomethacin), are detoxified by addition of glucuronic acid moieties by UDP-glucuronosyltransferase (UGT) in the liver and excreted in the bile *via* the conjugate export pump multidrug resistance-associated protein 2 (Mrp2; Treinen-Moslen and Kanz, 2006). In the gut, the glucuronic acids are enzymatically cleaved from drug conjugates by microbial *β*-glucuronidase, encoded by the inducible *gus* gene (Little et al., 2018). All major bacterial phyla present in the mammalian GI tract (*Bacteroidetes*, *Firmicutes*, *Proteobacteria*, *Actinobacteria*, *Clostridium*, and *Bifidobacterium)* express the *gus* gene (Pollet et al., 2017; Currò, 2018). However, *β*-glucuronidase activity varies depending on the bacterial species and the anatomical location along the small intestine (Mani et al., 2014). The reactivation of previously detoxified NSAIDs conjugates *via* enterohepatic circulation plays an important role in the pathogenesis of NSAID-induced enteropathy. Enterohepatic recirculation of NSAID determines repeated and prolonged exposures of the intestinal mucosa to relatively higher concentrations of the active molecules (Reuter et al., 1997; Seitz and Boelsterli, 1998; Boelsterli et al., 2013; Liang et al., 2015; Zhong et al., 2016).

Studies with *β*-glucuronidase inhibitors further support the hypothesis that enterohepatic circulation is an important factor in the development of NSAID-induced enteropathy. A small molecule inhibitor of bacterial *β*-glucuronidase, Inh1, can reduce the number and size of intestinal ulcers in mice treated systematically with diclofenac, without modifying the pharmacokinetics of diclofenac (LoGiudice et al., 2012). Similarly, Inh1 alleviates ketoprofen-or indomethacin-induced enteropathy in mice, without interfering with the biliary excretion of NSAID conjugates (Saitta et al., 2014). Inh1 is specific for *β*-D-glucuronidase and it does not affect mammalian orthologue *β*−glucuronidases (which are essential for proper lysosomal storage) and does not kill bacteria or mammalian cells (Wallace B. D. et al., 2010), indicating that the bacterial form is the major target (LoGiudice et al., 2012). Moreover, intestinal bacterial enzymes, including *β*-glucuronidase and 7*α*/*β*-dehydroxylase, are involved in the deconjugation and dehydroxylation of primary bile acids, synthetized by the liver, into the more cytotoxic secondary bile acids. NSAID-induced changes in the microbiota can elevate secondary bile acid ratio, favoring intestinal damage (Blackler et al., 2015). Furthermore, bacterial enzymes that produce large quantities of secondary bile acids can as well amplify the damage against the intestinal mucosa by increasing the enterohepatic circulation of NSAIDs (Duggan et.al., 1975; Seitz and Boelsterli, 1998; Zhou et al., 2010). Thus, the severity of NSAID enteropathy is correlated with the amount of drug excreted in the bile and the rate of enterohepatic circulation (Duggan et al., 1975). Indeed, ligation of the bile duct prevents NSAID-induced intestinal damage in mice and in rats (Yamada et al., 1993; Jacob et al., 2007). Moreover, intestinal damage by diclofenac is prevented in rats lacking the hepatocanalicular conjugate export pump, a protein required for the excretion of conjugated NSAIDs into the bile (Seitz and Boelsterli, 1998). Finally, the use of NSAIDs that do not undergo enterohepatic recirculation is not being associated with enteropathies (Reuter et al., 1997; Somasundaram et al., 1997).

In summary, these studies provide clear evidence that the complex pathogenesis of NSAID-induced enteropathy is determined by a combination of NSAID-topical effects, COX inhibition, drug–host–microbiota interconnectedness, enterohepatic recirculation of the drug, and the effect of secondary bile acids.

## Therapeutic Interventions Targeting the Gut Microbiota

The gut microbiota represents a target for therapeutic intervention since altering its functionality and metabolic capability may improve NSAID efficacy and/or to reduce NSAID side effects. Specifically several studies have reported the beneficial effects of targeting the gut microbiota against NSAID-induced small intestinal injury (Lanas and Scarpignato, 2006; Otani et al., 2017).

Some poorly absorbable antibiotics that target Gram-negative bacteria prevent NSAID-induced enteropathy in mice (Uejima et al., 1996; Koga et al., 1999; Watanabe et al., 2008a; Colucci et al., 2018) and in humans (Bjarnason et al., 1992; Davies et al., 1993; Scarpignato et al., 2017). However, these treatments are inconsistently effective in limiting intestinal damage (Syer et al., 2015). Furthermore, the use of antibiotics for extended periods of time may be deleterious since it could increase the risk of the development of multi-drug resistant bacteria and/or antibiotic-associated enteritis. Consequently, alternative agents or modes to alter the gut microbiota could represent better candidates for preventing or treating NSAID-induced enteropathy (Steidler, 2003; Mani et al., 2014; Syer et al., 2015; Gallo et al., 2016).

Supplementation with probiotics (rational selection of specific probiotic strains) in chronic users of NSAIDs may help to restore an altered intestinal microbiota (Mani et al., 2014, **Table 4**). Pre-treatment with viable *Lactobacillus casei strain Shirota* (LcS) improves indomethacin-induced enteropathy by suppressing of neutrophil infiltration and gene expression of inflammatory cytokines (Watanabe et al., 2009). Similarly, L-lactic acid produced by LcS suppresses indomethacin-induced small intestinal damage in rats (Watanabe et al., 2009). Moreover, culture supernatants of *Lactobacillus acidophilus* or *Bifidobacterium adolescentis* reduce NSAID-induced ileal damage by repressing unbalanced growth of aerobic bacteria and lipid peroxidation in rats (Kinouchi et al., 1998). Furthermore, the administration of *Bifidobacterium adolescentis* or *Faecalibacterium prausnitzii* prior naproxen treatment results in a significant reduction of the intestinal damage in rats, probably through an effect on the biosynthesis of cytoprotective short-chain fatty acids (Syer et al., 2015). Recently, it has been reported that the combination of lactoferrin with *Bifidobacterium longum* protects against diclofenac induced-enteropathy in rats partially by modulating the TLR-2/-4/NF-*κ*B pathways (Fornai et al., 2020).

**Table 4:** *In vivo* studies reporting the effect of probiotics on NSAID-induced enteropathy.

NSAID	Intervention	Specie	Outcome	Reference
Indomethacin (10 mg/kg; p.o.)	*Lactobacillus casei* strain Shirota	Rat	*↓* small intestinal injury and intestinal inflammation	Watanabe et al., 2009
5-bromo-2-(4-fluorophenyl)-3-(4-methylsulfonylphenyl) thiophene (BFMeT; 1,000 mg/kg; o.s.)	Culture supernatant of *Lactobacillus acidophilus* or *Bifidobacterium adolescentis*	Rat	*↓* reduction of ileal ulcer formation	Kinouchi et al., 1998
Naproxen (20 mg/kg, p.o.)	*Biﬁdobacterium adolescentis or Faecalibacteriaum prausnitzii*	Rat	*↓* severity small intestinal ulceration	Syer et al., 2015
Diclofenac (4 mg/kg, BID, p.o)	*Bifidobacterium Longum* plus Lactoferrin	Aged rat (males)	*↓* length of ileum lesions *↓* intestinal inflammation *↑* hemoglobin	Fornai et al., 2020
Aspirin (100 mg daily, p.o.) plus omeprazole (20 mg daily, p.o.)	*Lactobacillus Casei*	Human	*↓* number of mucosal breaks *↓* capsule endoscopy score	Endo et al., 2011
Low dose aspirin daily (p.o.)	*Lactobacillus gasseri*	Human	*↓* number of mucosal breaks *↓* reddened lesions	Suzuki et al., 2017
Aspirin (300 mg, daily, p.o)	*Bifidobacteriumbreve*	Human	*↓* number intestinal lesions *↓* intestinal inflammation	Mortensen et al., 2019
Indomethacin (50 mg, p.o.)	VSL#3	Human	*↓* intestinal inflammation	Montalto et al., 2010
Indomethacin (50–75 mg, p.o.)	*Lactobacillus plantarum*	Human	No effect of intestinal permeability	Mujagic et al., 2017
Indomethacin (75 mg, p.o.)	*Lactobacillus GG*	Human	No effect on intestinal permeability	Gotteland et al., 2001

So far, few studies have been performed in humans to investigate whether modulation of the gut microbiota with probiotics is an effective therapeutic approach against NSAID-induced enteropathy, and the results of these studies are discordant (Montalto et al., 2013). *Lactobacillus casei* significantly decreases the number of intestinal mucosal lesions in patients in the low-dose aspirin group compared to those in the control group (Endo et al., 2011). Furthermore, the administration of yogurt containing *Lactobacillus gasseri* reduces aspirin-induced small bowel injuries and mitigates GI symptoms in a double blind study in patients (Suzuki et al., 2017). A probiotic mixture consisting of eight different live bacteria (VSL#3) significantly decreases fecal calprotectin concentration, a marker of intestinal inflammation and enteropathy, in healthy volunteers treated with indomethacin compared to those treated with placebo in a randomized double-blind, placebo controlled cross-over trial (Montalto et al., 2010). *Bifidobacterium breve* protects against aspirin induced small-intestinal damage in a randomized, double-blind trial of healthy volunteers (Mortensen et al., 2019). On the contrary, *Lactobacillus plantarum* strains did not improve the intestinal permeability altered by indomethacin in a small randomized placebo controlled cross-over study in healthy volunteers (Mujagic et al., 2017). Similarly, ingestion of live *Lactobacillus GG* reduces alteration of the integrity of the gastric, but not the intestinal, mucosal barrier induced by indomethacin in healthy subjects (Gotteland et al., 2001).

In addition to the use of probiotics, rebamipide, a mucosal protective agent clinically used for treating gastritis and peptic ulcers, can prevent NSAID-induced small intestinal damage and improve intestinal healing mainly by regulating the intestinal microbiota in animals (Mizoguchi et al., 2001; Diao et al., 2012; Tanigawa et al., 2013; Kurata et al., 2015; Lai et al., 2015) and in humans (Niwa et al., 2008; Fujimori et al., 2011; Mizukami et al., 2011; Kurokawa et al., 2014; Watanabe et al., 2015; Ota et al., 2016; **Table 5**). Several mechanisms mediate the protective effect of rebamipide against NSAID small intestinal injuries, including its ability to upregulate alpha-defensin 5 gene and protein expression in the ileal tissue, which increases the abundance of Gram-positive bacteria and reduces Gram negative microbes, as reported in mice (Tanigawa et al., 2013).

**Table 5:** *In vivo* studies reporting the effect of rebamipide on NSAID-induced enteropathy.

NSAID	Intervention	Specie	Outcome	Reference
Indomethacin (10 mg/kg, s.c.)	Rebamipide (30–300 mg/kg, p.o.)	Rat	*↓* intestinal damage *↓* oxidative stress *↓*bacterial translocation	Mizoguchi et al., 2001;
Diclofenac (2.5 mg/kg, p.o.)	Rebamipide (100–400 mg/kg, p.o.)	Mouse	*↓* intestinal permeability *↓* oxidative stress *↑* inter-cellular tight junctions	Diao et al., 2012;
Indomethacin (10 mg/kg, p.o.)	Rebamipide (100 or 300 mg/kg, p.o.)	Mouse	*↓* intestinal damage *↑ Lactobacillales* *↓ Bacteroides* and *Clostridium* *↑* α-defensin 5 gene expression	Tanigawa et al., 2013;
Aspirin (200 mg/kg, p.o.)	Rebamipide (320 mg/kg, p.o.)	Mouse	*↓* intestinal damage *↑* COX-2, β-catenin, and c-myc gene expression *↑* PGE_2_ level	Lai et al., 2015;
Indomethacin (10 mg/Kg, p.o.)	Rebamipide (30 and 100 mg/Kg, p.o.)	Rat	*↓* intestinal damage *↓ Enterococcaceae* and *Enterobacteriaceae* *↓* mucosal inflammation	Kurata et al., 2015
Diclofenac (75 mg, p.o.)	Rebamipide (300 mg p.o.)	Human	*↓* small-intestinal mucosal injury	Niwa et al., 2008
Diclofenac (75 mg, p.o.)	Rebamipide (300 mg p.o.)	Human	No effect on small-intestinal mucosal injury	Fujimori et al., 2011
Low-dose aspirin (p.o.)	Rebamipide (300 mg p.o.)	Human	*↓* mucosal breaks on the ileum	Mizukami et al., 2011
Low-dose aspirin and/or NSAID (p.o.)	Rebamipide (300 mg p.o.)	Human	*↓* small-intestinal mucosal injury	Kurokawa et al., 2014
Enteric coated aspirin (100 mg, p.o.)	Rebamipide (900 mg p.o.)	Human	*↓* mucosal breaks *↓* intestinal damage	Watanabe et al., 2015
Enteric coated aspirin (100 mg, p.o.)	Rebamipide (300 or 900 mg p.o.)	Human	No effect on intestinal lesions and intestinal inflammation	Ota et al., 2016

Additionally, as discussed earlier, inhibition of the hydrolytic cleavage of NSAID conjugates by targeting bacterial *β*-glucuronidase with small molecule reagents could represent an alternative effective intervention to improve NSAID safety profiles (Boelsterli et al., 2013).

In summary, therapeutic intervention targeting the gut microbiota is a promising approach to prevent NSAID-induced small intestinal injury, but additional data are needed from larger clinical long term longitudinal studies to assess its clinical benefits. Thus, well-designed trials taking in consideration physical activity and eating habits of the volunteers and time of administration of the probiotic should be performed to evaluate the actual role of agents targeting the microbiota to prevent NSAID enteropathy. This will also help to clarify the eventual differences among probiotic strains, dose-response relationships, and the optimal duration of therapy.

## Limitations/Future Studies

In this review, we have discussed studies that provide insight into the reciprocal NSAID–gut microbiota interactions in animal models and in humans. Such interactions are identified mostly through studies using germ-free mice and animals treated with antibiotic cocktails or colonized with specific bacterial consortia. Additional evidence for NSAID–gut microbiota interconnectedness may arise from metagenomics-based associations, microbiota environment-mimicking culture-based experiments, biochemical characterization of gut microbiota enzymes, and functional studies.

The investigation of the microbiota in animal models often fails to predict the results obtained in humans. This is due to many factors, including species-specific differences in gastrointestinal anatomy, physiology and microbiota profiles; reduced microbiota diversity and dynamics due to laboratory conditions; and genetic similarity of used rodent strains (Nguyen et al., 2015). However, the use of traditional animal models in microbiota studies allows perturbations of the intestinal microbial taxa (*i.e.* abiotic and gnotobiotic animals) that are necessary to help in assessing causality of the complex host-microbiota interconnectedness and in developing mechanistic hypotheses. Indeed, there are several examples of gut microbiota–drug interactions characterized in mice that have been further validated in humans, including studies with digoxin (Saha et al., 1983; Haiser et al., 2013) and proton pump inhibitors (Hung et al., 2015; Imhann et al., 2016).

Some of the studies discussed in this review are limited by the fact that they often focus on fecal metagenomics. The luminal content and different anatomical intestinal locations are characterized by significantly different microbial communities that also play a role into the host–gut microbiota metabolic interactions. Moreover, *16S ribosomal RNA* sequencing profiles provide information only on the bacteria present in the gut microbiota and not on nonbacterial members of the gut community, like fungi, archaea, and viruses, which are also candidate modulators of drug metabolism and govern the dynamic microbiota composition and functionalities. Therefore, a holistic analysis that includes the archaea, the virome, the phageome, and the mycobiome may provide a more complete picture of host–microorganism-drug interconnectedness. Indeed, fungi and parasites can metabolize AA and produce immunomodulatory lipid mediators, including PGE_2_, PGD_2_ and leukotrienes, some of which modulates microbial fitness during pathogenesis (Noverr et al., 2001; Noverr et al., 2002; Hervé et al., 2003; Tsitsigiannis et al., 2005; Valdez et al., 2012; Kim et al., 2014).

## Concluding Remarks

The investigation of the impact of selective and nonselective NSAIDs on host/gut microbiota interactions provide new insights into the pharmacology and metabolism of NSAIDs and into the molecular mechanisms contributing to their efficacy and toxicity. Despite the important consequences of this interconnectedness for the host, the specific gut microbial strains, genes, and metabolic pathways that mediate NSAID disposition, efficacy and toxicity are still poorly understood. It still remains a challenge to link microbial biotransformation to specific enzymes and to elucidate their biological effects. Thus, further studies to improve our understanding of how host–gut microbiota and NSAIDs interact are needed to identify individual factors that influence the outcome of NSAID therapy.

## Author Contributions

DM and ER critically reviewed the literature and wrote the manuscript. DM and ER approved the submitted version.

## Conflict of Interest

The authors declare that the research was conducted in the absence of any commercial or financial relationships that could be construed as a potential conflict of interest.
